# The Hepatoprotective Effects of *Moringa oleifera* against Antiretroviral-Induced Cytotoxicity in HepG_2_ Cells: A Review

**DOI:** 10.3390/plants12183235

**Published:** 2023-09-12

**Authors:** Mbasakazi Saki, Helena De Villiers, Claudia Ntsapi, Charlette Tiloke

**Affiliations:** Department of Basic Medical Sciences, School of Biomedical Sciences, Faculty of Health Sciences, University of the Free State, Bloemfontein 9300, South Africa; 2015061019@ufs4life.ac.za (M.S.); devilliershc@ufs.ac.za (H.D.V.); ntsapimc@ufs.ac.za (C.N.)

**Keywords:** HIV/AIDS, tenofovir, reactive oxygen species, antioxidants, *Moringa oleifera*, human HepG_2_ liver cells

## Abstract

The untreated human immunodeficiency virus (HIV), a lentivirus species that attacks immune cells (CD4+ T cells), causes acquired immunodeficiency syndrome (AIDS). HIV-positive people manage HIV/AIDS by using antiretroviral therapy (ART). The ART treatment regimen contains two nucleoside reverse transcriptase inhibitors (NRTIs) and one non-nucleoside reverse transcriptase inhibitor/integrase strand transfer inhibitor. Tenofovir, an NRTI approved for managing HIV infection, is associated with hepatic steatosis and lactic acidosis, which are linked to mitochondrial toxicity and oxidative stress. Due to side-effects associated with ART, people living with HIV often use medicinal plants or a combination of medicinal plants with ART to promote adherence and diminish the side-effects and cytotoxicity. The *Moringa oleifera* (MO) tree from the family of Moringaceae is among the medicinal trees studied in managing HIV/AIDS in sub-Saharan Africa. The MO tree extracts have been reported to have inhibitory activity primarily against HIV due to their bioactive compounds. However, there is a scarcity of knowledge about the use of the MO tree amongst HIV/AIDS patients receiving ART in South Africa and its effect on patient compliance and outcomes. Thus, this review aims to outline the impact of MO aqueous leaf extract on oxidative stress and antioxidant responses in human HepG_2_ liver cells after exposure to antiretrovirals such as tenofovir. The review will contribute to a comprehensive understanding of the potential protective effect of MO aqueous leaf extract on tenofovir-induced cytotoxicity.

## 1. Introduction

Acquired Immune Deficiency Syndrome (AIDS), caused by the Human Immunodeficiency Virus (HIV), is a highly communicable disease that continues to impose a significant burden on national healthcare systems across the world but specifically in sub-Saharan Africa [1]. Globally, more than 30 million HIV-positive people have lost their lives following the identification of the first HIV-positive patient [2]. However, since the availability of antiretroviral therapy (ART) in the mid-1990s, the number of HIV/AIDS-related deaths has steadily declined [3].

In South Africa (SA), the accepted standard of care for HIV treatment includes using a combination of three active drugs, namely Tenofovir–Lamivudine–Dolutegravir (TLD) [4]. The TLD treatment provides rapid viral suppression and a high genetic barrier to drug resistance [5]. Consequently, the use of TLD treatment has shifted HIV infection from a terminal illness to a long-term, manageable chronic disease. People living with HIV (PLHIV) can have a life expectancy similar to that of HIV-negative individuals [6]. Unfortunately, some patients receiving ART may develop severe side-effects such as drug-induced liver injury [1]. Due to the side-effects associated with ART, PLHIV, especially in the rural areas of SA, tend to use traditional remedies such as medicinal trees/plants to ameliorate the side-effects associated with the use of ART [7].

The *Moringa oleifera* (MO) tree from the family of Moringaceae is one such example of a medicinal plant; it has been used for centuries in traditional medicine [8]. MO’s traditional uses include healing skin infections, wounds, fever, diarrhea, and sore throats [9]. The MO tree is widely used due to its high concentration of phytochemicals that work synergistically to induce their medicinal effects [8]. Scientifically, MO is documented to possess anti-inflammatory, antihypertensive, antimicrobial, antioxidant, anti-diabetic, and antiviral effects [10]. In addition, MO has been shown to improve renal and hepatic functions [11]. Various parts of the MO tree (i.e., flowers, seeds, roots, and leaves) contain a wide range of bioactive compounds, including flavonoids and phenolic acids [12]. However, the leaves contain the most significant amount of bioactive compounds and therefore have a wide range of medicinal properties such as anti-inflammatory, anticancer, and antioxidant [12,13]. Even though there is evidence to support the health benefits associated with MO treatment, little is known about the use of the MO leaf extracts amongst HIV/AIDS patients receiving ART in SA and its effect on patient compliance and outcomes. Hence, this review focuses on exploring the potential hepatoprotective effect of MO leaf extract after exposure to ART, such as tenofovir.

Research methodology: A literature review on in vitro-based research studies investigating the hepatoprotective effects of MO aqueous leaf extracts against drug-induced oxidative stress was conducted.

Inclusion criteria for the literature review:

- Literature published in English.
- Literature published at least 5 years ago.
- African-based research study articles.
- Published literature on HIV, antiretroviral therapy, oxidative stress, antioxidants, MO, and its bioactive compounds.
- Published literature intended to investigate the underlying mechanism of action.
- Published literature with rationale and scientific evidence.
- Research studies identified through scientific databases such as Google Scholar, PubMed, and Science Direct.
- Biorender.com for creating figures.

Exclusion criteria for the literature review:

- Conference papers.
- Bioactive compounds not related to the antioxidant effect of MO.
- Research studies that are not focusing on the HepG_2_ cell line.

## 2. Prevalence of HIV/AIDS

AIDS was first discovered in patients in the United States of America in 1981 [14]. It is caused by the human immunodeficiency virus 1 (HIV-1) and 2 (HIV-2) [15]. AIDS originated from two species of lentivirus [16], which entered the human population through cross-species transmission in the early twentieth century [17]. These viruses are spread by one of three modes of transmission: sexual, parenteral, and mother-to-child [18].

Globally, the vast majority (~98%) of HIV infections are caused by HIV-1 [14], a variant of HIV that primarily attacks the immune system’s CD4+ T cells [19]. The CD4+ T cells are thymus lymphocytes that recognize antigenic peptides in the form of MHC class II molecules [20]. These cells help B cells produce antibodies and are required to generate cytotoxic and memory CD8+ T cells that destroy infected cells [21]. However, when infected by HIV, CD4+ T cells replicate the virus [22]. HIV thus hijacks and manipulates the transcriptional and translational machinery of CD4+ T cells to replicate itself [23].

Worldwide, the number of HIV-1-infected people continues to increase. There are approximately 40 million HIV-positive people worldwide, with developing countries accounting for 95% of those infected [2]. Globally, SA has the largest number of HIV-1 infections [24]. Since the estimated 5.3 million PLHIV reported in 2004 [25], there has been an increase in HIV-1 infections in SA. Approximately 7.5 million PLHIV, an estimated 200,000 new HIV-1 infections, and 74,000 HIV/AIDS-related mortalities were reported in 2019 [24]. The prevalence of HIV is approximately 19.5% in the South African adult population [26]. Poverty, a lack of education about the virus and its modes of transmission, a high incidence of rape, the non-disclosure of HIV-positive status to partners, and mother-to-child HIV transmission are overarching factors that can be associated with the increasing number of HIV-1 infections in SA [27,28].

**Figure 1:**
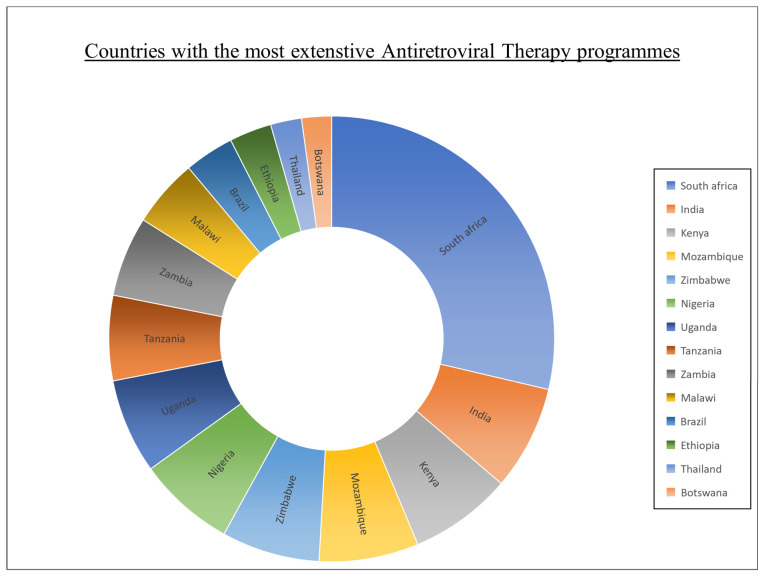
Countries with the most extensive antiretroviral treatment programs (created by researcher, M. Saki, in 2023 [19]).

In 2004, SA started the national rollout of ART to treat HIV-1 infections [25]. Since 2005, the percentage of deaths related to HIV/AIDS decreased from 50.8% to 31.1% in 2016 [29]. This decrease is primarily attributed to the government’s rapid scale-up of public-sector HIV resources to make ART extensively accessible [29]. As of 2016 (see Figure 1), SA reportedly has the most extensive ART program, with approximately 3.9 million individuals receiving antiretrovirals (ARVs) [19]. This value is four times the number of all other ART programs globally, equating to 24% of all ART programs worldwide [19].

The program’s success is expected to increase as plans and policies initiated in 2004 have improved HIV patient outcomes and survival rates throughout the country [29]. The plans and policies include the provision of ART free of charge and the use of community-based programs expanding on HIV care and treatment services [29]. At first, only those with CD4+ T cell counts of 200 cells μL^−1^ or less were approved for treatment [30]. A low CD4+ T cell count indicates that the immune system has been compromised by HIV and/or the disease is progressing [31]. However, in 2016, SA implemented the Universal Test and Treat (UTT) program. All individuals who tested positive were allowed to receive treatment regardless of their CD4+ T cell count [30].

**Figure 2:**
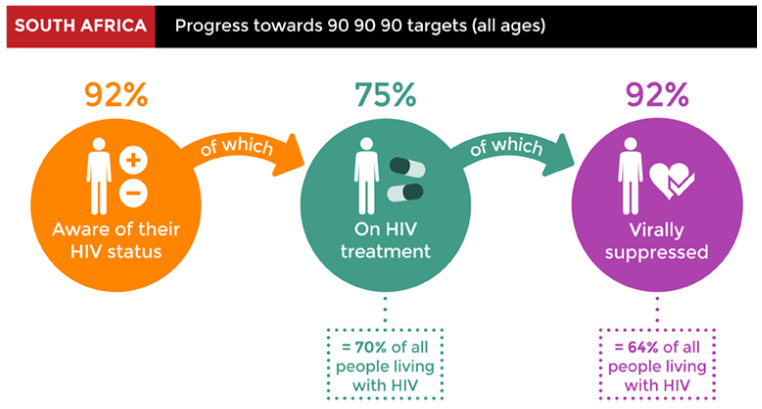
The progress toward the 90–90–90 targets [33].

The initiation of the UTT program led to the Joint United Nations Programme on HIV/AIDS (UNAIDS) 90–90–90 mantra, which was adopted in 2016. In simple terms, it means that 90% of the populace should be aware of their HIV status, 90% of those who are aware of their HIV status should be on therapy, and 90% of those receiving treatment should achieve suppressed viral loads [32]. According to the UNAIDS 2020 report, 92% of people in SA are aware of their HIV status, 75% receive therapy, and 92% are virally suppressed (see Figure 2) [33].

To manage the HIV epidemic, it is critical to achieve optimum virologic suppression across key population groups [34]. Using ART is the only proven management strategy for HIV-1 infection that has improved the quality of life in PLHIV [35].

### 2.1. Antiretroviral Therapy

**Figure 3:**
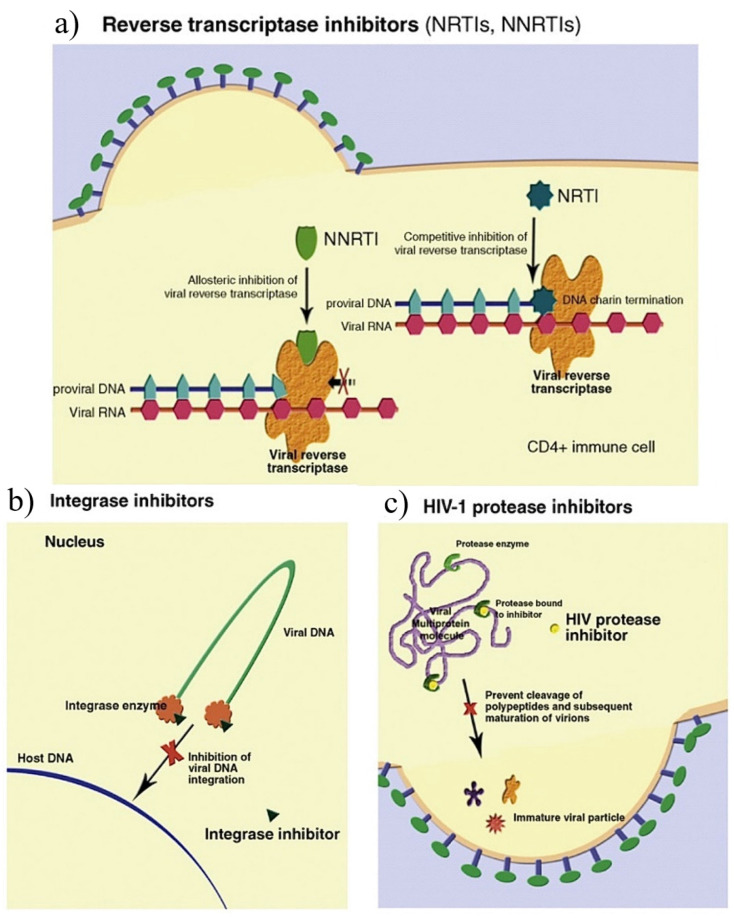
Mechanism of action of antiretroviral therapy drugs: (**a**) reverse transcriptase inhibitors (NRTIs, NNRTIs); (**b**) integrase inhibitors; and (**c**) HIV-1 protease inhibitors [36].

There are five ART drug categories approved by the USA Food and Drug Administration (FDA), which include NRTIs, non-nucleoside reverse transcriptase inhibitors (NNTRIs), integrase strand transfer inhibitors (InSTIs), protease inhibitors (PIs), and entry inhibitors [3]. Each class targets a specific phase of the HIV-1 replication cycle (see Figure 3) [19].

The NRTIs halt the elongation of the proviral deoxyribonucleic acid (DNA) strand by blocking the HIV-1 reverse transcriptase (RT) enzyme responsible for converting viral ribonucleic acid (RNA) into viral DNA [37,38]. The NNRTIs inhibit DNA polymerase activity by inducing a conformational change, disrupting the enzyme’s catalytic site [39]. The HIV integrase enzyme, responsible for integrating viral DNA into the host cell’s DNA, is inhibited by InSTIs [36]. The PIs are responsible for inhibiting HIV-1 protease, which cleaves newly synthesized polyproteins (Gag and Gag-Pol) into a mature infectious virus [40,41].

### 2.2. The Approved Standard Treatment for HIV

The approved standard treatment for HIV-1 consists of a combination of three drugs from at least two different categories [4]. One of the first NRTIs accepted to treat HIV-1 infection was zidovudine (AZT). Following its acceptance, several other NRTIs, such as tenofovir, was produced and used in combination with NNRTIs or PIs [42]. Until 2018, the first-line regimen for HIV-1 treatment in many countries was the combination of Tenofovir–Lamivudine–Efavirenz (TLE); that is, two NRTIs (Tenofovir–Lamivudine) and one NNRTI (Efavirenz) [43]. However, TLE has a low genetic barrier to drug resistance and causes neuropsychiatric side-effects [44]. Subsequently, in 2019, the South African National Department of Health changed the prescribed standard initial treatment from TLE to Tenofovir–Lamivudine–Dolutegravir (InSTI) (TLD), a fixed-dose combination [45].

### 2.3. The Types of Antiretroviral Drugs

The backbone for HIV-1 treatment, TLD, targets a specific phase of the HIV-1 replication cycle, aggressively suppresses viral replication, and halts the progression of HIV-1 infection [46]. The TLD treatment is more tolerable and has a high genetic barrier to drug resistance [6].

#### 2.3.1. Tenofovir

Tenofovir is a nucleotide analog of adenosine 5′-monophosphate [47]. It is a highly hydrophilic compound with two negative charges resulting in low intestinal membrane permeability [48]. To improve oral bioavailability and membrane permeability, tenofovir is commercially available as a pro-drug, tenofovir disoproxil fumarate (TDF) [49]. Following oral administration, TDF is rapidly converted to tenofovir in the intestinal walls through esterase hydrolysis [50]. Tenofovir then enters cells through organic anion transporters 1 and 3 [51].

Intracellularly, tenofovir is phosphorylated by adenylate kinases and subsequently phosphorylated by nucleoside diphosphate kinases into its active form, tenofovir diphosphate [52]. Tenofovir diphosphate is an analog of deoxyadenosine-5′-triphosphate (dATP), a common substrate for DNA polymerase. Tenofovir diphosphate terminates the viral DNA chain elongation by competing with dATP to be incorporated into viral DNA [53]. The kidneys excrete Tenofovir through glomerular filtration and tubular secretion [51]. Organic anion transporters in the basolateral membrane actively transport about 20–30% of tenofovir into renal proximal tubule cells. Subsequently, tenofovir is secreted into the tubular lumen by the apical membrane transporters and multidrug-resistance proteins, MRP-4 and MRP-2 (encoded by ABCC4 and ABCC2 genes, respectively) [53].

#### 2.3.2. Lamivudine

Lamivudine (3TC) forms part of the NRTIs; it inhibits viral DNA synthesis via RT DNA chain termination post phosphorylation [37]. 3TC is highly soluble and rapidly absorbed, with a bioavailability ranging from 82 to 86% for oral administration [5]. Intracellularly, 3TC is metabolized to its active triphosphate form, lamivudine triphosphate (3TC-TP), through kinase phosphorylation [54].

The 3TC-TP competes with the corresponding endogenous nucleoside triphosphate, deoxycytidine triphosphate (dCTP), for binding to the viral RT. Once incorporated into the viral DNA, chain termination results due to the absence of a 3′-hydroxy (3′-OH) group to enable the 3′-5′-phosphodiester linkages essential for DNA elongation [55]. The majority of 3TC is eliminated through filtration and active renal tubular secretion. Metabolism is a minor route of elimination, with only 10% of the parent drug metabolized to an inactive trans-sulfoxide metabolite that is excreted in the urine [5].

#### 2.3.3. Dolutegravir

Dolutegravir (DTG), an orally bio-available integrase strand transfer inhibitor, is an efficacious, well-tolerated drug with a high barrier to drug resistance [56]. Upon oral administration, DTG binds and inhibits the active site of integrase, an HIV enzyme that catalyzes the integration of viral DNA into chromosomal DNA, leading to viral replication [57]. DTG is metabolized in the liver by uridine 5′-diphosphate-glucuronosyltransferase (UGT) 1A1 and cytochrome P450 (CYP) 3A4 [58].

### 2.4. Antiretroviral Drugs’ Side-Effects

The most common side-effects of ART-TLD-based regimens include nausea, diarrhea, hypoglycemia, insomnia, and headaches [59]. Appetite loss, vomiting, diarrhea, and abdominal pain caused by ART use can result in malnutrition [60]. HIV and malnutrition can be detrimental to the immune system, decreasing the number of CD4+ and CD8+ T cells. A compromised immune system will subsequently increase the body’s susceptibility to opportunistic infections, including pneumocystis pneumonia, cryptococcal meningitis, and mycobacterium tuberculosis [60,61]. Opportunistic infections and malnutrition can worsen disease progression and increase HIV-related mortality [60].

**Figure 4:**
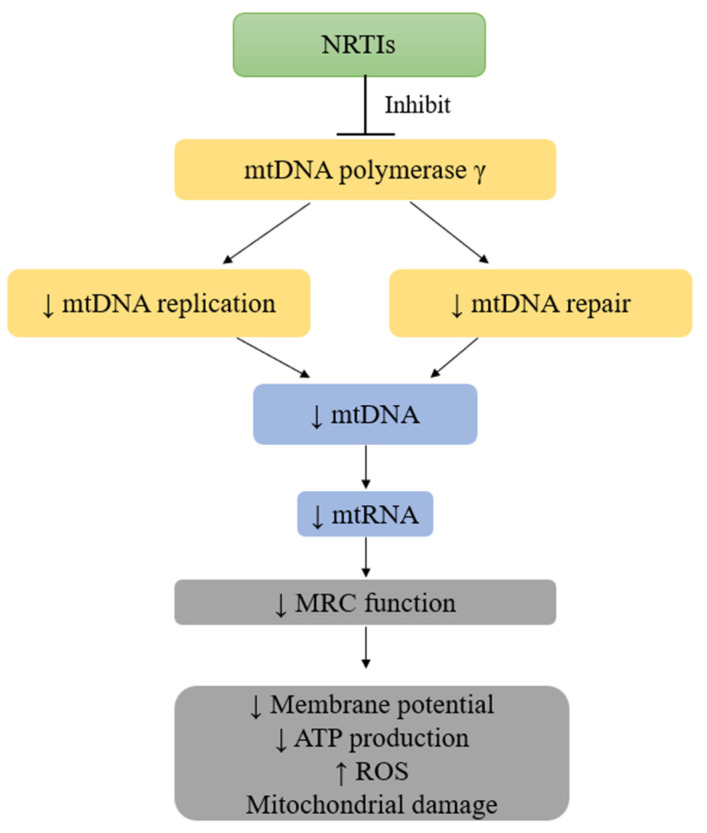
Mechanism of ARV-drug-induced hepatotoxicity. ATP: adenosine triphosphate; MRC: mitochondrial respiratory chain; ROS: reactive oxygen species; ↑: increase; ↓: decrease (created by the researcher, M. Saki, in 2023 [66]).

Reports from preclinical- and clinical-based studies have also linked ART with hepatotoxicity associated with oxidative stress [62]. Hepatotoxicity is a liver injury or impairment of liver function caused by exposure to xenobiotics such as drugs, alcohol, peroxidized fatty acids, environmental toxicants, and even some medicinal plants [63]. Hepatotoxicity may include hepatitis, granuloma, lactic acidosis, cholestasis, and hepatic steatosis [64]. The TDF regimen has been associated with severe lactic acidosis and hepatic steatosis [49]. The mechanism proposed behind TDF causing the latter complications is the inhibition of mitochondrial DNA (mtDNA) polymerase gamma (γ) [65]. Mitochondrial toxicity can manifest as nephrotoxicity, myopathy, pancreatitis, peripheral neuropathy, lactic acidosis, and hepatic steatosis [65]. Figure 4 illustrates the mechanism of ART-induced cytotoxicity in liver cells.

Figure 4 demonstrates that ARVs such as NRTIs inhibit DNA polymerase γ and lead to decreased mtDNA, loss of mitochondrial membrane potential, and oxidative phosphorylation, consequently precipitating oxidative stress [67]. NRTIs lack a 3′-OH group on their pentose rings, having nucleoside as their base. Due to the missing 3′-OH group, the NRTIs prevent the formation of the 3′-5′-phosphodiester bonds in growing DNA chains and terminate mtDNA chain elongation [68]. As a result, mtDNA copy numbers decrease, as well as mitochondrial-encoded genes, which are essential components of the mitochondrial respiratory chain (MRC) function. This leads to a disrupted electron transport chain and a concomitant reduction in membrane potential and ATP production by the mitochondrion. This destruction in mitochondrial function can result in increased ROS production and changes in mitochondrial morphology [66,69].

### 2.5. Oxidative Stress

Oxidative stress is an imbalance between the excessive generation of free radicals or reactive oxygen species (ROS) and their eradication by the antioxidant defense system [70]. Free radicals are atoms with an unpaired electron in their outer orbital [71]. Free radicals are unstable and highly reactive; however, they gain stability by attracting electrons from other compounds. The compound loses an electron and becomes a free radical, triggering a chain reaction cascade, ultimately damaging the living cell [72]. The term “reactive oxygen species” refers to any oxygen-containing molecule (radical or non-radical) capable of causing harmful reactions. These include the superoxide anion (O_2_ˉ), hydrogen peroxide (H_2_O_2_), hydroxyl radical (HO•), alkoxyl radical (RO), peroxyl radical (RO_2_), hydroperoxyl radical (HO_2_), hypochlorous acid (HOCl), and singlet oxygen (O_2_) [73].

During the oxidative stress reaction (illustrated below), the formation of superoxide results from the one-electron reduction of O_2_, the disproportionation of two superoxide molecules yields H_2_O_2_ and O_2_, and the oxidation of ferric iron by H_2_O_2_ yields HO• and the hydroxide anion [73]:

O_2_ + e^−^ → O_2_^−^ (Superoxide)

2O_2_^−^ + 2H+ → O_2_ + H_2_O_2_ (Hydrogen peroxide)

Fe^2^ + H_2_O_2_ → Fe^3^ + HO• + OH^−^ (Hydroxyl radical)

**Figure 5:**
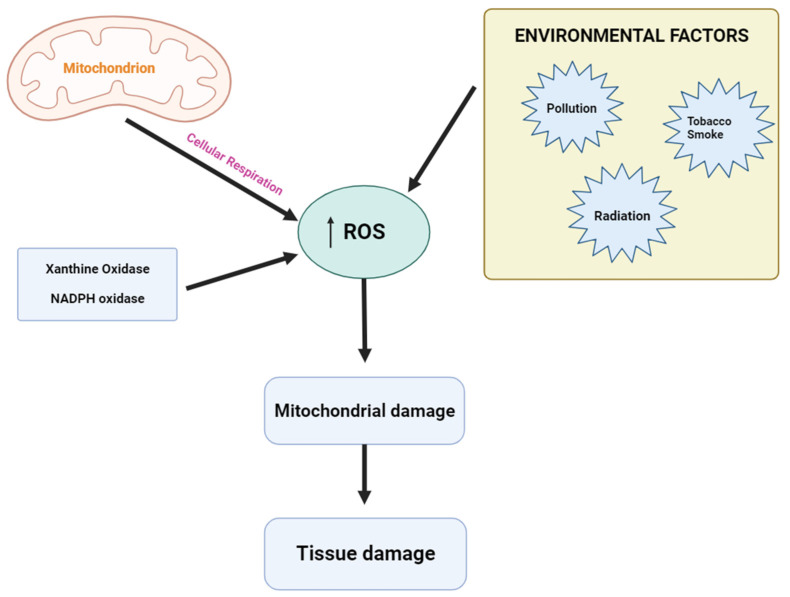
Oxidative stress. Multiple factors induce oxidative stress by upregulating (↑) ROS (created by the researcher, M. Saki, in 2023 using Biorender.com).

As illustrated in Figure 5, free radicals/ROS are formed due to adenosine triphosphate (ATP) production by the mitochondria when cells utilize oxygen to produce energy [74].

In addition to mitochondria, ROS are produced by various enzymes, such as NADPH oxidases and xanthine oxidase (XO) [75]. The sum of ROS produced by metabolic processes can be induced by environmental stimuli in the form of various stresses, including pollution, tobacco smoke, alcohol, transition metals, heavy metals, pesticides, industrial solvents, drugs such as ARVs, paracetamol, halothane, and radiation among others [76].

ROS include not only O_2_ˉ, H_2_O_2_, and HO•, but also a group of nitrogen-containing molecules called reactive nitrogen species (RNS) [77]. The nitroxyl anion, nitrosonium cation, higher oxides of nitrogen, S-nitrosothiols, and dinitrosyl iron complexes are all examples of RNS [78]. Another prominent effect of ROS is lipid peroxidation, which occurs when membrane phospholipids are brought into contact with an ROS oxidizing agent [75]. Lipid peroxidation is a process in which free radical species remove electrons from lipids. Subsequently, the lipids become reactive free radicals that can propagate lipid peroxidation chain reactions [79]. Lipid peroxidation forms several oxidation products, including lipid hydroperoxides (LOOH) and aldehydes such as malondialdehyde (MDA) and 4-hydroxynonenal (4-HNE) [80].

Among the aldehydes produced through lipid peroxidation, MDA has gained the most attention, given that MDA is made at high levels during lipid peroxidation and is commonly used as a measure of oxidative stress [81]. MDA is widely used as a biomarker for lipid peroxidation because of its ready reaction with thiobarbituric acid [82]. Uncontrolled free radicals/ROS production occurs when antioxidants (e.g., glutathione, superoxide dismutase, catalase, and vitamins) are saturated due to aging, stress, physical damage, or pathological disease [83].

### 2.6. Antioxidants

Antioxidants are compounds that scavenge free radicals/ROS and intracellularly retain a more reduced redox state [84]. The antioxidant defense system comprises endogenous and exogenous antioxidants [85]. Endogenous antioxidants produced by the body are divided into enzymatic and non-enzymatic antioxidants [86]. Enzymatic antioxidants include superoxide dismutases (SODs), catalases (CATs), and glutathione peroxidases (GPxs). Non-enzymatic antioxidants include polyamides, linolenic acid, bilirubin, albumin, uric acid, glutathione, transferrin, ceruloplasmin, and coenzyme Q10 [85]. Exogenous antioxidants are vitamins A, C, and E, selenium (Se), carotenoids, and polyphenols [87].

Antioxidants can directly decrease oxidative damage by accepting or donating an electron to eliminate the unpaired condition of the radical [88]. Antioxidants can also indirectly reduce free radicals by inhibiting the activity or expression of free-radical-generating enzymes. Examples of free-radical-generating enzymes are NADPH oxidase and XO [88]. Another function associated with antioxidants is increasing the activity or expression of intracellular enzymatic antioxidants such as SOD, CAT, and GPx [88]. SOD, CAT, and GPx are the first-line antioxidant enzymes that suppress or prevent the formation of free radicals/ROS. These enzymes are known to neutralize any molecule with the potential of developing into a free radical or any free radical with the potential to induce the production of other radicals [89]. The expression of SOD, CAT, and GPx is regulated by the non-coding DNA sequence antioxidant response element (ARE). ARE is activated by nuclear-factor-erythroid-2-related factor 2 (Nrf2) [90].

#### 2.6.1. Superoxide Dismutase

**Figure 6:**
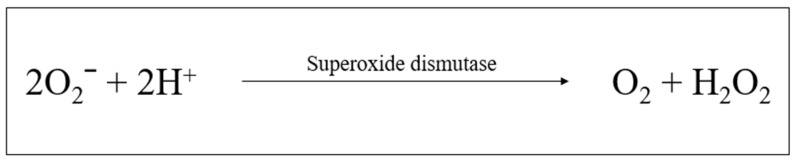
Superoxide dismutase enzyme reaction (created by the researcher, M. Saki, in 2023).

Superoxide dismutases (SODs) are a group of metalloenzymes that decrease O_2_^−^ levels by catalyzing the dismutation of the O_2_^−^ free radical into molecular oxygen and H_2_O_2_ (see Figure 6) [91]. They require a metal cofactor for their antioxidant activity. In this regard, SODs are classified into three forms, including iron SOD (Fe-SOD), manganese-dependent SOD (Mn-SOD), and copper-zinc-SOD (Cu/Zn-SOD) [89]. Superoxide dismutase 2 (SOD2) is an Mn-SOD. The SOD2 gene mapping encodes it to chromosome 6, which is present in prokaryotes and mitochondria of eukaryotes [89]. The SOD2 is a vital component of the antioxidant defense system against mitochondrial superoxide radicals [92].

#### 2.6.2. Catalase

Catalase (CAT) is a homotetramer in which each monomer (62.5 kDa) contains a haem B (also known as protoheme IX) group responsible for the enzymatic activity [93]. The CAT participates in the dismutation of H_2_O_2_ to oxygen and water in a two-step reaction [94]. The first step of the reaction involves the formation of compound I, the product of the reaction of H_2_O_2_ with catalase haem (Reaction (1)). Subsequently, compound I is decomposed upon reaction with a second H_2_O_2_ molecule in the catalytic reaction, releasing oxygen and water (Reaction (2)) [95]:

Compound I: catalase−Fe^3+^ + H_2_O_2_ → catalase−FeO^3+^ + H_2_O (1)

Catalytic reaction: catalase−FeO^3+^ + H_2_O_2_ → catalase−Fe^3+^ + O_2_ + H_2_O

In sum: 2H_2_O_2_ → O_2_ + 2H_2_O (2)

#### 2.6.3. Glutathione

Glutathione (GSH) is a thiol tripeptide (γ-glutamyl cysteinyl glycine) comprising three amino acids (glutamic acid, cysteine, and glycine) [96]. The synthesis of GSH from cysteine, glutamate, and glycine is catalyzed sequentially by two cytosolic enzymes, glutamate cysteine ligase (GCL) and GSH synthetase [97]. GCL catalyzes the first of two ATP-dependent steps in GSH synthesis, the formation of γ-glutamylcysteine (γ-GC) from glutamate and cysteine. The second step is catalyzed by GSH synthetase, which joins glycine to γ-GC, thus forming GSH [98].

GCL consists of two separately coded proteins, a catalytic subunit (GCLC) and a modifier subunit (GCLM) [99]. It has been shown that GSH production is paralleled with GCLC gene expression, which is primarily regulated at the transcription level. The GCLC gene is shown to have oxidative-stress-responsive elements in the promoter/enhancer region. Several *cis*-acting DNA elements contribute to the transcriptional up-regulation of the GCLC gene in response to oxidative stress, providing a protective mechanism against oxidative stress [100].

**Figure 7:**
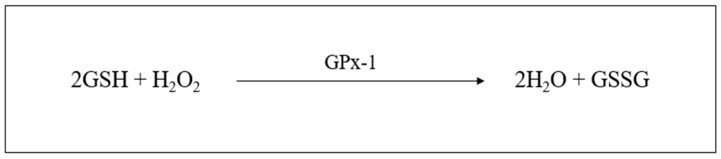
Glutathione peroxidase enzymatic reaction 1 (created by the researcher, M. Saki, in 2023).

In all mammalian tissues, GSH is the most abundant non-protein thiol that protects against oxidative stress [101]. The antioxidant function of GSH is primarily accomplished through GSH peroxidase-1 (GPx-1)-catalyzed reactions, reducing H_2_O_2_ to water and lipid peroxides to their corresponding alcohols, mainly in the mitochondria and cytosol [89]. The primary reaction that GPx-1 (selenocysteine (Sec)-containing enzyme) catalyzes is 2GSH + H_2_O_2_ → GSSG + 2H_2_O (see Figure 7) [102].

**Figure 8:**
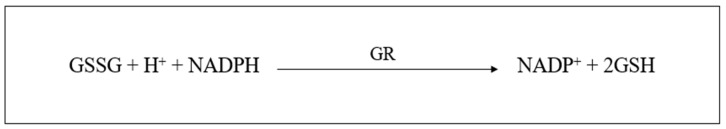
Glutathione peroxidase enzymatic reaction 2 (created by the researcher, M. Saki, in 2023).

Reducing equivalents come from two equivalents of GSH, which are oxidized to glutathione disulfide (GSSG). For the maintenance of free radical detoxification in a cell, GSSG needs to be converted to GSH by the glutathione reductase (GR) enzyme using nicotinamide adenine dinucleotide phosphate (NADPH) (see Figure 8) [103].

#### 2.6.4. Nuclear-Factor-Erythroid-2-Related Factor 2

**Figure 9:**
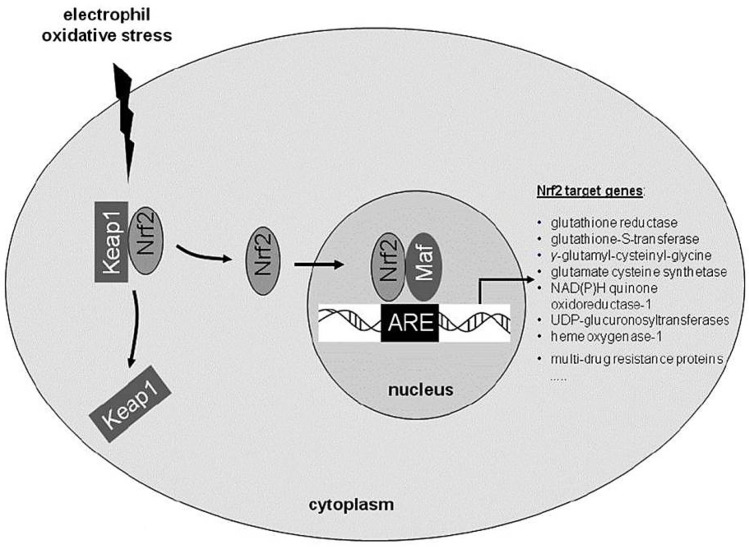
Activation of the antioxidant transcription factor, nuclear-factor-erythroid-2-related factor 2 [107].

The expression of most antioxidant enzymes is tightly regulated by the antioxidant response element (ARE), a non-coding DNA sequence activated by nuclear-factor-erythroid-2-related factor 2 (Nrf2) [90]. The Nrf2, a nucleus transcription factor bound to its inhibitor, Kelch-like ECH-associated protein (Keap1), is a vital transcription factor regulating cellular redox homeostasis [104]. Following exposure to oxidants (see Figure 9), Nrf2 is dissociated from Keap1 through oxidation of the cysteine residues of the Nrf2–Keap1 complex [105]. Nrf2 translocates to the nucleus and binds to ARE in the upstream regulatory regions of genes, encoding for detoxification and antioxidant enzymes, thereby leading to enhanced transcription [106].

### 2.7. The Use of Traditional African Medicinal Plants

An estimated 72% of the Black South African population uses traditional medicines for primary healthcare needs [108]. This can be ascribed to several factors, including easy access to medicinal plants, low costs, and extensive knowledge and expertise within the local communities [109]. There are about ten prominently used South African medicinal plants, which include *Aspalathus linearis* (Fabaceae), *Agathosma betulina* (Rutaceae), *Aloe ferox* (Asphodelaceae), and *Hypoxis hemerocallidea* (African potato) [110]. Each medicinal plant contains a wide range of diverse bioactive compounds and high levels of phytochemicals, which act as natural antimicrobial, anticancer, antispasmodic, antipyretic, antioxidant, and antiviral agents in the human body [7,111]. South African medicinal plants have also shown efficacy in treating hypertension, heartburn, arthritis, rheumatism, type 2 diabetes mellitus, gastrointestinal disturbances, menstrual difficulties, headache, heartburn, and gout [110]. PLHIV frequently uses traditional African medicines (ATMs) with Western medications, including ARVs [35]. The ATMs may diminish the side-effects of ARVs and their cytotoxicity and promote treatment adherence [35]. However, research studies evaluating the potential herb–drug interactions in a clinical setting are still warranted.

Medicinal plants that traditional medicine practitioners most extensively use in Sub-Saharan Africa for adjuvant HIV/AIDS treatment and related disorders are *Hypoxis hemerocallidea* (African potato), *Sutherlandia frutescens* (Cancer bush) [112], and MO [7]. In SA, the African potato, together with the cancer bush, is considered one of the two most popular medicinal plants used to boost the immune system of HIV patients [113,114]. Despite the popularity of their use and the support of the Ministries of Health and NGOs in certain African nations, no clinical trials are investigating the efficacy and limited evidence of harm for the potential of drug interactions with antiretroviral drugs [113]. MO is considered a highly nutritive tree in many parts of Africa and Asian countries [115]. The various health benefits of MO, particularly its leaves, are well researched, documented, and confirmed in several studies. MO’s most well-studied and exploited uses are medicinal and nutritional [115].

A Nigerian study by Gambo and colleagues in 2021 showed that MO leaf powder supplementation increased the CD4+ T cell count of PLHIV on ART (TLE) [60]. This can be attributed to MO’s nutraceutical benefits [60]. A study by Monera-Penduka and colleagues in 2017 showed that MO was well tolerated when taken with nevirapine by HIV patients. MO inhibits the CYP3A4 enzyme, which is responsible for metabolizing nevirapine. However, the safety profile of nevirapine was not altered when co-administered with MO [116]. The MO tree extracts have also shown inhibitory activity, specifically against HIV-1, Herpes Simplex Virus (HSV), and Hepatitis B Virus (HBV), which damages the liver by causing inflammation, cirrhosis, and liver cancer [7]. However, there is limited information about the use of MO amongst HIV/AIDS patients receiving TLD in SA and its effects on patient compliance and outcomes.

*Moringa oleifera* is a medicinal tree from the family of Moringaceae, commonly found in Asia and Africa, including Nigeria, Namibia, Ghana, Senegal, and SA [12,60,117]. In SA, MO is farmed in several provinces, including Gauteng, Limpopo, Mpumalanga, and KwaZulu-Natal [115]. The MO tree is known for its anthelmintic, antiseptic, detergent, anti-ulcerogenic, anti-inflammatory, anti-microbial, antioxidant, anti-hyperglycaemic, anti-clastogenic, anticancer, and anti-fibrotic effects [8]. For centuries, many cultures worldwide have used MO to treat skin infections, blackheads, anxiety, anemia, asthma, bronchitis, catarrh, chest congestion, cholera, and many other illnesses [118].

Ayurvedic, the traditional Indian system of medicine, is an ancient yet living tradition that is equal to conventional Western medicine and traditional Chinese medicine [119]. It is based on drug discovery, whereby therapeutically active ingredients are first identified based on ethnic uses and then verified through clinical trials. It is a holistic healing system based on over 7000 plants and about 8000 remedies, all of which have been documented [120]. The traditional Ayurvedic system of medicine shows that MO can prevent approximately 300 diseases, and its leaves have been exploited for preventive and curative purposes [121].

### 2.8. Moringa oleifera

**Figure 10:**
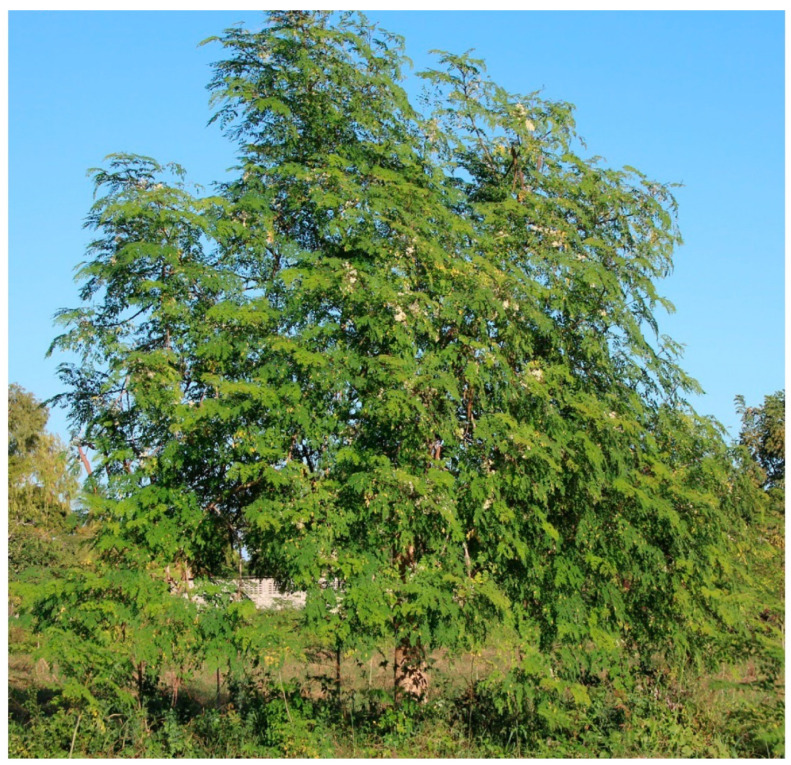
*Moringa oleifera* tree [121].

*Moringa oleifera,* commonly known as the ‘Drumstick’ or horseradish tree, is a small, soft-wooded deciduous tree with sparse foliage cover (see Figure 10) [122]. MO is a fast-growing, highly drought-tolerant, and multi-purpose tree. It is usually 5–10 m tall but can grow up to 15 m [123]. MO belongs to the monogeneric family of shrubs and trees, Moringaceae, considered to have its origin in Agra and Oudh, in the northwest region of India and south of the Himalayan Mountains. It is now cultivated throughout the Middle East, in almost the whole tropical belt, and it was introduced in Eastern Africa from India at the beginning of the 20th century [124]. The tree has been scientifically classified accordingly into the Plantae kingdom, Magnoliophyta division, Magnoliopsida class, Brassicales order, Moringaceae family, *Moringa* genus, and *oleifera* species [125]. This plant is widely used as a nutritional herb and contains valuable pharmacological actions like antiasthmatic, antidiabetic, hepatoprotective, anti-inflammatory, anticancer, and antioxidant [126].

MO trees excel mainly in tropical and sub-tropical regions and are known to thrive in a wide range of soil types, mostly heavy clay and waterlogged, with a pH between 4.5 and 8, at an altitude of up to 2000 m [121,124]. It thrives in dry to moist tropical or subtropical climes, with an annual precipitation of 760 to 2500 mm [121]. Furthermore, among all climatic factors that affect plant growth, temperature is one of the most important factors governing natural geographical plant distribution, tree performance, physiology, and productivity [124]. The tree requires between 25 and 35 °C to have optimal growth and a high production of pods and leaves, resulting in its most cost-effective cultivation [127].

**Figure 11:**
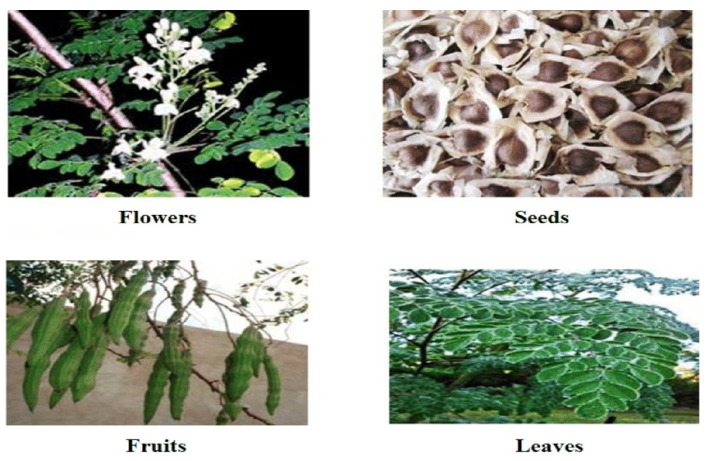
Constituents of the *Moringa oleifera* tree [126].

Various parts of the tree (Figure 11) consist of numerous bioactive components, including vitamins, polyphenols, isothiocyanates, tannins, and saponins [12]. The roots, bark, gum, leaf, flowers, fruit (pods), seed, and seed oil of the MO tree have various biological activities that protect against gastric ulceration and hypertension, in addition to anti-diabetic and anti-inflammatory effects [10].

The leaves are the most used part of the tree for nutritious and medicinal purposes. The leaves contain the most extensive amounts of vitamins C and A; flavonoids, including myricetin, quercetin, rutin, and phenolic acids; and carotenoids, such as lutein, β-carotene, and zeaxanthin [13]. The high content of bioactive compounds provides several health advantages, including antidiabetic, anticancer, anti-inflammatory, and antioxidant properties. The antioxidant effect of MO leaves is mostly attributed to flavonoids, phenolic acids, and carotenoids [128]. MO leaves predominantly contain quercetin (43.75%) and equal percentages (18.75%) of other flavonoids. The concentration of flavonoids varies with the environmental conditions. MO leaves harvested in South Africa and Namibia had 17 distinct flavonoids, with quercetin (35%), kaempferol (35%), isorhamnetin (24%), and apigenin (6%) derivatives, but leaves harvested in sub-Saharan Africa contained just 12 different flavonoids [129]. MO leaves contain gallic acid as their major phenolic acid. Ferulic acid, ellagic acid, caffeic acid, o-coumaric acid, and chlorogenic acid are also detected in the leaves [9]. Gallic acid is the most abundant, with a concentration of 1.034 mg/g of dry weight [12]. The leaves have abundant carotenoids with a total amount varying from 44.30 to 80.48 mg/100 g on a fresh weight basis among eight different cultivars [129].

#### 2.8.1. Flavonoids

**Figure 12:**
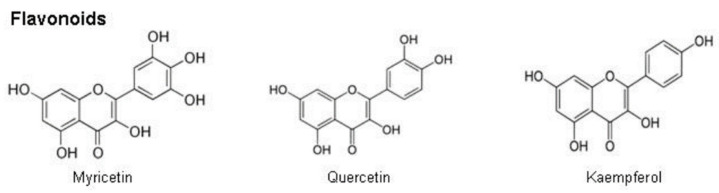
Chemical structures of flavonoids [121].

Flavonoids are polyphenolic phytochemicals found in fruits, vegetables, and grains [130]. Intake of flavonoids has been shown to protect against chronic diseases associated with oxidative stress. The main flavonoids (see Figure 12) found in MO leaves are myricetin, quercetin, and kaempferol, in concentrations of 5.8, 0.207, and 7.57 mg/g, respectively [12]. Flavonoids are a group of diphenyl propane compounds (C6-C3-C6). They have the general structure of a 15-carbon skeleton composed of two phenyl rings (A and B), linked through a heterocyclic pyran or pyrone ring in the middle [131].

**Figure 13:**
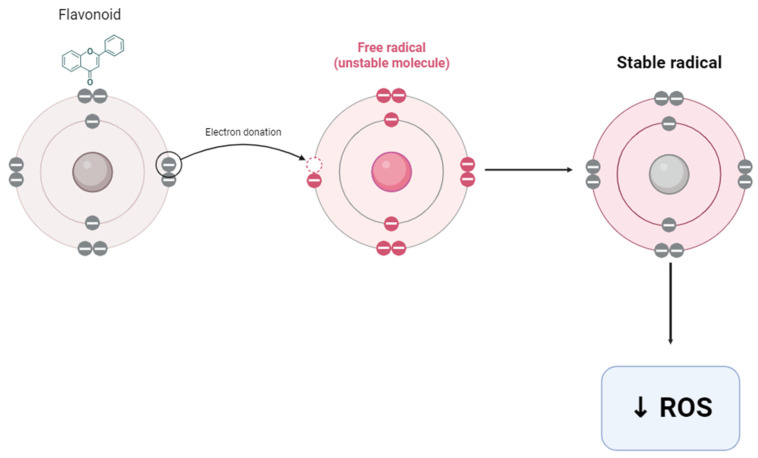
Antioxidant effect of flavonoids. Flavonoids decrease (↓) ROS by donating electrons to free radicals (created by the researcher, M. Saki, in 2023 using Biorender.com).

The potent antioxidant activity exhibited by flavonoids in vitro is primarily due to their ability to trap free radicals via the metal chelation and donation of electrons or hydrogen atoms (see Figure 13) [131]. Flavonoids are oxidized by free radicals, resulting in a more stable, less-reactive radical [132].

#### 2.8.2. Phenolic Acids

**Figure 14:**
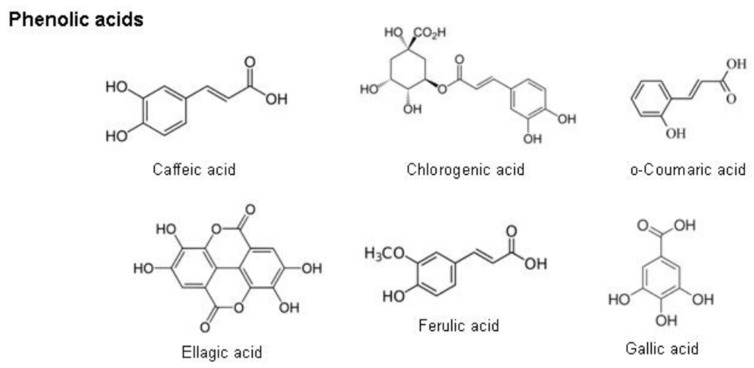
Chemical structures of phenolic acids [121].

Phenolic acids are a sub-group of phenolic compounds derived from hydroxybenzoic acid and hydroxycinnamic acid, naturally present in plants [121]. Caffeic acid, ellagic acid, ferulic acid, and chlorogenic acid are found in the phenolic acid group (Figure 14). Phenolic acids are distributed ubiquitously in plants and play a significant protective role in oxidative stress conditions [133].

**Figure 15:**
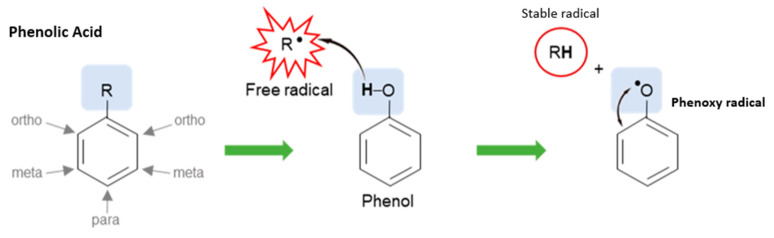
Antioxidant effect of phenolic acids (created by the researcher, M. Saki, in 2023 [135]).

Phenolic acids possess antioxidant activity due to their chemical nature: hydroxyl groups attached to the pentyl ring. They stabilize free radicals by donating a hydroxyl group, forming a delocalized and stabilized unpaired electron, a phenoxy radical, across the phenolic ring (see Figure 15). The degree of antioxidant activity is determined by the position and number of the phenolic hydroxyl groups [134].

#### 2.8.3. Carotenoids

**Figure 16:**
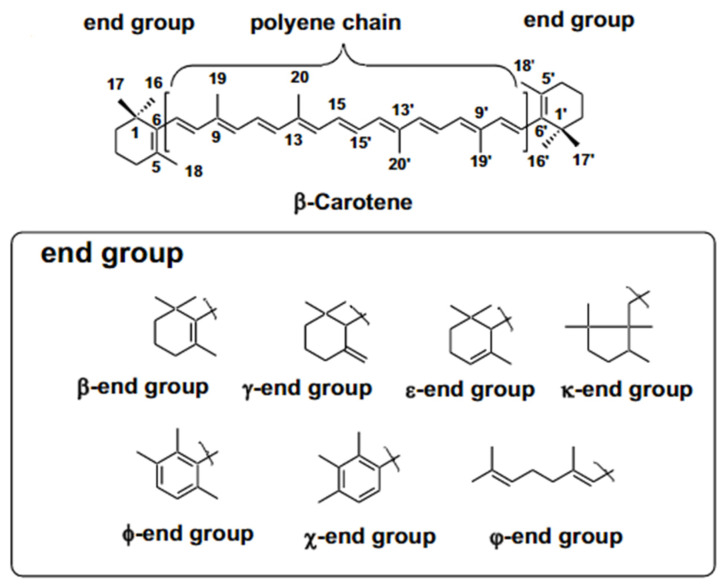
Basic structures of carotenoids and end groups [136].

Carotenoids are tetraterpene pigments, which exhibit yellow, orange, red, and purple colors [136]. Plants and some microorganisms such as bacteria, fungi, and yeasts produce these pigments. Among the carotenoids, the *β*-carotene is the most abundant in foods that have the highest activity of provitamin A [137]. Most carotenoids (Figure 16) consist of eight isoprene units with a 40-carbon skeleton. Their general structures commonly consist of a polyene chain with nine conjugated double bonds and an end group at both ends of the polyene chain [136].

**Figure 17:**
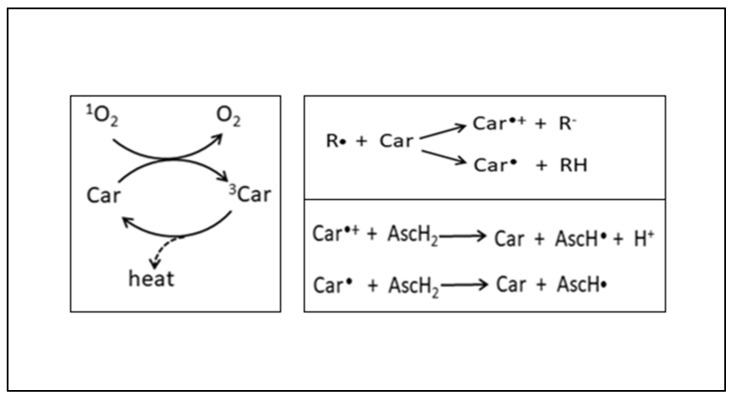
Antioxidant effect of carotenoids [139]. Reactions of carotenoids with singlet oxygen or radicals and regeneration by ascorbate. ^1^O_2_: singlet oxygen; O_2_: oxygen; Car: carotenoids; R•: radicals.

Carotenoids are well known for their ability to physically and chemically quench oxygen. They scavenge ROS and play a protective role in various ROS-mediated disorders, such as multiple forms of cancer, cardiovascular diseases, and neurological and eye-related disorders [138]. Carotenoids quench singlet oxygen, remove peroxy radicals, modulate carcinogen metabolism, inhibit cell proliferation, stimulate communication between cells, and increase the immune response (Figure 17) [137].

### 2.9. The Hepatoprotective Effects of Moringa oleifera against Oxidative Stress

**Figure 18:**
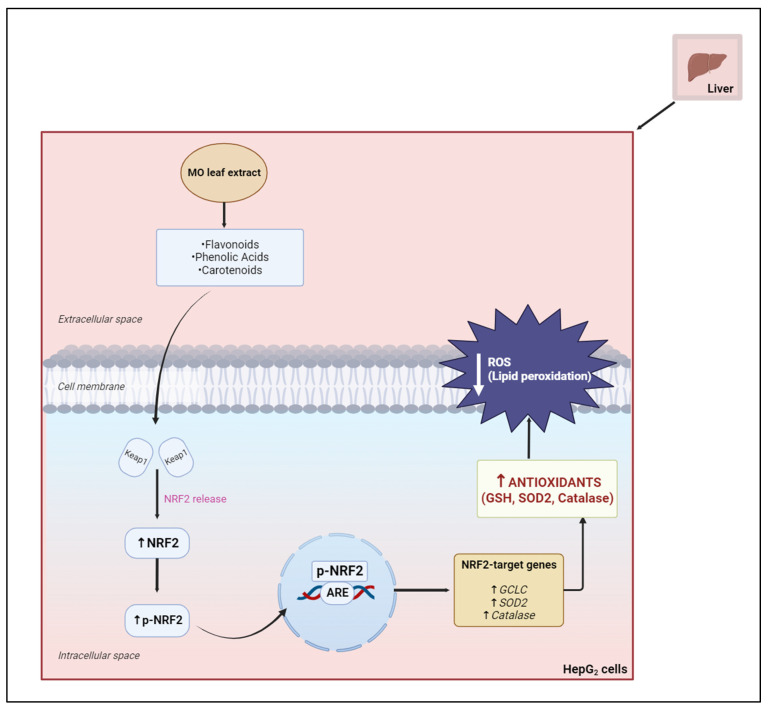
The hepatoprotective effects of MO against oxidative stress (created by the researcher, M. Saki, in 2023 using Biorender.com). The antioxidant effect of MO responds to increased ROS. During oxidative stress, MO activates the Nrf2-Keap1 complex and releases Nrf2. p-Nrf2, the active form of Nrf2, translates to the nucleus and binds to ARE, and promotes the transcription of antioxidant genes. This results in the upregulation of antioxidants and the downregulation of ROS, thus decreasing oxidative stress.

The MO aqueous leaf extracts contain large amounts of bioactive compounds or high levels of phytochemicals such as flavonoids, phenolic acids, and carotenoids, which act synergistically to induce their medicinal effects [140]. Daily consumption of MO is recommended. However, an overdose of MO may cause a high accumulation of iron. High iron can cause gastrointestinal distress and hemochromatosis. Hence, a daily dose of 70 g of MO is suggested to be suitable and prevents the over-accumulation of nutrients [141]. In vivo, the aqueous leaf extract, a rich source of antioxidant compounds, is responsible for protecting against oxidative-stress-induced diseases [126]. In vitro, studies have reported MO’s renal and hepatoprotective properties against several drugs, such as gentamicin, pyrazinamide, rifampicin, and acetaminophen, mainly attributable to its leaves [142]. To determine the potency of MO as a protective agent in vitro, a half-maximal inhibition concentration (IC_50_) will have to be determined. Scientists often use the HepG_2_ cell line as an in vitro model system that mimics the natural in vivo environment to assess hepatoxicity and the protective effects of aqueous leaf extracts (Figure 18) [143,144].

### 2.10. The Use of Human HepG₂ Liver Cells In Vitro

Currently, drugs used to treat several viral infections, such as HBV, HSV, or HIV, display consistent side-effects, including mitochondrial toxicity [145]. Several in vitro models and techniques have been developed to analyze the impact of such drugs. The HepG_2_ cells (derived from the human hepatoma) are an excellent model to investigate mitochondrial toxicity due to their high organelle content and mtDNA. They are extensively used by several investigators [145]. The HepG_2_ cell line was established from a liver tumor biopsy obtained from a 15-year-old Caucasian male in the 1970s [11]. It is the most frequently used hepatoma cell line in the testing and research on drug-induced liver injury [11].

Several scientists have researched ART using the HepG_2_ cell line. In 2017, Paemanne and colleagues used the HepG_2_ cell line to investigate the effect of the nevirapine (NVP) regimen on mitochondrial dysfunction. The study results showed that NVP induces mitochondrial dysregulation in HepG_2_ cells [146]. Shamsabadi, 2014 investigated the hepatotoxic effects of the components of Atripla and Eviplera on HepG_2_ cells. The results showed that NRTIs, tenofovir, and emtricitabine had no hepatotoxic effects in vitro compared to the NNRTIs, efavirenz, and rilpivirine [147]. A research study by Nagiah and colleagues in 2015 has also established the appropriate application of this cell line as an in vitro model to evaluate drug metabolism or the toxicity of antiretroviral drugs, including tenofovir. Further research studies that take on a similar approach to assess tenofovir-induced cytotoxicity and incorporate MO treatment to evaluate the hepatoprotective effects will be beneficial.

## 3. Conclusions

To date, various biological activities of different parts of the MO plant have been reported. However, no in vitro studies have assessed the hepatoprotective effects of MO aqueous leaf extract against ART-induced cytotoxicity in liver cells. Therefore, further research is warranted to investigate the effect of MO aqueous leaf extract on oxidative stress and antioxidants.

## 4. Value of This Study

This study is of scientific, clinical, and public value. It contributes to the scientific knowledge by determining the extent to which the MO tree can commonly be used for medicinal purposes and its potential to minimize tenofovir-induced oxidative stress in human HepG_2_ liver cells. To achieve this, the use of various experimental techniques such as spectrophotometry, luminometry, ELISA, Western blotting, and qPCR is employed. Societal disparities that contribute to inadequate access to basic healthcare and the occurrence of malnutrition for a majority of low-income households result in the use of the MO tree. The use of the MO tree for the provision of medicine, food, as well as skin and hair supplements may serve as a promising alternative, more so for people residing in the rural areas of SA. HIV-infected people often use MO with ART in an attempt to mitigate side-effects, boost their immune system, and improve their health. However, using medicinal plants with drugs, including ART, mainly in Africa and other developing countries, is not well researched and thus poorly regulated. Therefore, more studies evaluating the efficacy of medicinal plants in relation to reducing the cytotoxicity associated with ART use are needed. Thus, results from this study will provide insight and broaden our understanding of how the bioactive compounds present in MO aqueous leaf extract may have a hepatoprotective effect against tenofovir-induced oxidative stress in human HepG_2_ liver cells.

## Abbreviations

AIDS: Acquired immunodeficiency syndrome; ARE, Antioxdiant response element; ART, Antiretroviral therapy; ATP, Adenosine triphosphate; ATMs, Traditional African medicines, AZT, Zidovudine; CAT, Catalase; Cu/Zn-SOD, Copper-zinc-SOD; CYP, cytochrome P450; dATP, deoxyadenosine-5′-triphosphate; dCTP, deoxycytidine triphosphate; DNA, deoxyribonucleic acid; DTG, Dolutegravir; FDA, USA Food and Drug Administration; Fe-SOD, Iron SOD; GCL, Glutamate cysteine ligase; GCLC, Glutamate cysteine ligase catalytic subunit; GCLM, Glutamate cysteine ligase modifier subunit; GPx, Glutathione peroxidase; GSH, Glutathione; GSSG, Glutathione disulfide; HIV, Human immunodeficiency virus; H_2_O_2_, Hydrogen peroxide; HO_2_, hydroperoxyl radical; HO•, hydroxyl radical; HOCl, hypochlorus acid; InSTI, integrase strand transfer inhibitors; LOOH, lipid hydroperoxides; MDA, Malondialdehyde; mDNA, mitochondrial DNA; MO, *Moringa oleifera*; Mn-SOD, Manganese-dependent SOD; NADPH, Nicotinamide adenine dinucleotide phosphate; Nrf2, Nuclear-factor-erythroid-2-related factor 2; NNRTIs, Non-nucleoside reverse transcriptase inhibitors; NRTIs, Nucleoside reverse transcriptase inhibitors; O_2_, singlet oxygen; O_2_ˉ, anion; PLHIV, People living with HIV/AIDS; PIs, protease inhibitors; RNA, Ribonucleic acid; RNS, Reactive nitrogen species; RO, alkoxyl radical; RO_2_, peroxyl radical; ROS, Reactive oxygen species; Se, Selenium; SOD, Superoxide dismutase; TDF, Tenofovir disoproxil fumarate; TLD, Tenofovir–Lamivudine–dolutegravir; TLE, Tenofovir–lamivudine–efavirenz; UGT, 5′-diphosphate-glucuronosyltransferase; UNAIDS, Joint United Nations Programme on HIV/AIDS; UTT, Universal test and treat; XO, Xanthine oxidase; 3′-OH, 3′-hydroxy; 3TC, Lamivudine; 3TC-TP, lamivudine triphosphate; 4-HNE, 4-hydroxynonenal γ, gamma; γ-GC, γ-glutamylcysteine.
